# Supplementary material for: Combining Historical and Molecular Data to Study Nearly Extinct Native Italian Grey Partridge (Perdix perdix) at the Turn of the Twentieth Century
Source: Biology (Basel). 2024 Sep 10;13(9):709. doi: 10.3390/biology13090709 (PMC11429232; doi:10.3390/biology13090709)
Supplement: Supplementary file 1 [file biology-13-00709-s001.zip › biology-3163342-supplementary.pdf]

Supplementary files

Summary

TABLE S1 2

TABLE S2 3

TABLE S3 10

BOX S1 11

BOX S2 13

BOX S3 14

Table S1

List of the 15 museums and 5 private collections that provided historical samples of grey partridge. For each one, the number of samples and the collection period is indicated.

**Museum name**	**N° samples**	**period**
Civic Museum of Zoology - Roma	26	1892-1927
Museum Malmerendi - Faenza	8	1948-1971
Museum of La Specola - Firenze	15	1876-1949
Museum of Natural History and Territory - Pisa	10	1835-1923
Museum of Natural History and Territory Città della Pieve	1	unknown
Museum of Natural History of Friuli	4	1923-1935
Museum of Stazzano - Alessandria	15	1950-1999
Museum of the Accademia dei Fisiocratici - Siena	7	1884-1910
Museum of Zoology University of Bologna	4	1897-1907
Natural History Museum of Casalina - Perugia	3	1855-1857
Natural History Museum of Pavia	1	1887
Ornithological and Natural Science Museum of Ravenna	5	1915-1946
Ornithological Museum Foschi - Forlì	10	1933-1956
Regional Interdisciplinary Museum Palazzo d'Aumale, Sicily - Terrasini	11	1900-1954
Zoological Collection ISPRA Ozzano dell'Emilia - Bologna	6	1895-1949
Private Collection - Marche	4	1982
Private Collection - Piedmont	1	1950
Federcaccia - Reggio Calabria	1	1956
Federcaccia - Imola	2	1960-70
Private Collection - Umbria	6	unknown
Total	140	

Table S2

List of *Perdix perdix* haplotypes retrieved in the present work and original ID from grey literature and previously published studies from GenBank. *Accession number GB = GenBank identification code; Haplotype ID GeneBank = Haplotype name present in GB; Published haplotype = Unique long fragment haplotypes retrieved from other authors or collapsed due to length base pair reduction. New Haplotype = new code assigned based on the short fragment analyzed in this study. The * was used to associate captive or wild with the correct region of origin if there are more than two.*

Accession number GB	Haplotype ID GenBank	Published Haplotype	New Haplotype	Country of origin	Wild or captive	Reference
AF115404.1	ME	*P. p. lucida* ME_E6_E14	Pdx_E1	Ireland, Bulgaria	Wild	Liukkonen et al. 2002
-		E6	Pdx_E1	Finland	Wild	Liukkonen et al. 2002
-		E14	Pdx_E1	Greece	Wild	Liukkonen et al. 2002
AF115405.1	MW	*P. p. perdix* MW	Pdx_W1	Italy, Finland	Tuscany Captive, Wild	Liukkonen et al. 2002
AY601124.1	PPL2	ME_E6_E14	Pdx_E1	Finland	Captive	Liukkonen 2006
AY601125.1	PPL3	ME_E6_E14	Pdx_E1	Finland	Captive	Liukkonen 2006
AY601127.1	PPL5	ME_E6_E14	Pdx_E1	Finland	Captive	Liukkonen 2006
AY601129.1	PPL7	ME_E6_E14	Pdx_E1	Finland	Captive	Liukkonen 2006
AY601130.1	PPL8	ME_E6_E14	Pdx_E1	Finland	Captive	Liukkonen 2006
AY601131.1	PPL9	ME_E6_E14	Pdx_E1	Finland	Captive	Liukkonen 2006
AY601133.1	PPL11	ME_E6_E14	Pdx_E1	Finland	Captive	Liukkonen 2006
AY601134.1	PPL12	ME_E6_E14	Pdx_E1	Finland	Captive	Liukkonen 2006
AY601141.1	PPLC1	Pdx_GB_9U	-	Finland	Captive	Liukkonen 2006
AY601142.1	PPP1	Pdx_GB_6U	-	EU	Wild	Liukkonen 2006
AY601143.1	PPP2	Pdx_GB_7U	-	EU	Wild	Liukkonen 2006
AY601144.1	PPP3	-	Pdx_W1	EU	Wild	Liukkonen 2006
AY601145.1	PPP5	-	Pdx_W1	EU	Wild	Liukkonen 2006
AY601146.1	PPP4	Pdx_GB_8U	-	EU	Wild	Liukkonen 2006
AY601147.1	PPPC1	W2_W28	Pdx_W1	EU	Captive	Liukkonen 2006
AY601148.1	PPPC2	Pdx_GB_4U	-	EU	Captive	Liukkonen 2006
AY601149.1	PPPC3	W9_W10_W26	-	EU	Captive	Liukkonen 2006

AY601150.1	PPPC4	Pdx_GB_5	-	EU	Captive	Liukkonen 2006
AY601151.1	PPPC5	-	Pdx_W1	EU	Captive	Liukkonen 2006
AY601152.1	PPPC6	-	Pdx_W1	EU	Captive	Liukkonen 2006
AY601153.1	PPPC7	-	Pdx_W1	EU	Captive	Liukkonen 2006
D66891.1	-	-	Pdx_W1	EU	Unknow origin	Fumihito et al. 1995
GU827386.1	-	*P. p. italica* W17	-	Italy	Captive	Lucentini et al. 2010
JN817437.1	PPDK1	Pdx_GB_2		Denmark	Historical and wild	Andersen, Kahlert 2012
JN817438.1	PPDK1	Pdx_GB_14U	-	Denmark	Wild	Andersen, Kahlert 2012
JN817439.1	PPDK3	-	Pdx_W1	Denmark	Wild	Andersen, Kahlert 2012
JN817440.1	PPDK4	Pdx_GB_13	-	Denmark	Wild	Andersen, Kahlert 2012
JN817441.1	PPDK5	Pdx_GB_13	-	Denmark	Wild	Andersen, Kahlert 2012
JN817442.1	PPDK6	-	Pdx_W3	Denmark	Wild	Andersen, Kahlert 2012
JN817443.1	PPDK7	Pdx_GB_12U	-	Denmark	Wild	Andersen, Kahlert 2012
JN817445.1	PPDK9	Pdx_GB_11U	-	Denmark	Wild	Andersen, Kahlert 2012
JN817446.1	PPDKC10	-	Pdx_W1	Denmark	Captive	Andersen, Kahlert 2012
MH001731.1	Pdx_GB_15	unpublished	-	Greece	Wild	Kalaentzis, Triantafyllidis, Gagavouzis
MH001733.1	Pdx_GB_15	unpublished	-	Greece	Wild	Kalaentzis, Triantafyllidis, Gagavouzis
MH001736.1	Pdx_GB_10	unpublished	-	Greece	Wild	Kalaentzis, Triantafyllidis, Gagavouzis
MH001737.1	Pdx_GB_15	unpublished	-	Greece	Wild	Kalaentzis, Triantafyllidis, Gagavouzis
MH001738.1	Pdx_GB_15	unpublished	-	Greece	Wild	Kalaentzis, Triantafyllidis, Gagavouzis
MH001739.1	Pdx_GB_15	unpublished	-	Greece	Wild	Kalaentzis, Triantafyllidis, Gagavouzis
MH001740.1	Pdx_GB_10	unpublished	-	Greece	Wild	Kalaentzis, Triantafyllidis, Gagavouzis
MH001741.1	Pdx_GB_10	unpublished	-	Greece	Wild	Kalaentzis, Triantafyllidis, Gagavouzis
MH001742.1	Pdx_GB_10	unpublished	-	Greece	Wild	Kalaentzis, Triantafyllidis, Gagavouzis

MH001744.1	Pdx_GB_10	unpublished	-	Greece	Wild	Kalaentzis, Triantafyllidis, Gagavouzis
MH001696.1	GRF001	unpublished	Pdx_W1	Greece	Captive	Kalaentzis, Triantafyllidis, Gagavouzis
MH001697.1	GRF002	unpublished	Pdx_W1	Greece	Captive	Kalaentzis, Triantafyllidis, Gagavouzis
MH001698.1	GRF008	unpublished	Pdx_W1	Greece	Captive	Kalaentzis, Triantafyllidis, Gagavouzis
MH001699.1	GRF012	unpublished	Pdx_W1	Greece	Captive	Kalaentzis, Triantafyllidis, Gagavouzis
MH001700.1	GRF013	unpublished	Pdx_W1	Greece	Captive	Kalaentzis, Triantafyllidis, Gagavouzis
MH001701.1	GRF014	unpublished	Pdx_W1	Greece	Captive	Kalaentzis, Triantafyllidis, Gagavouzis
MH001702.1	GRF021	unpublished	Pdx_W1	Greece	Captive	Kalaentzis, Triantafyllidis, Gagavouzis
MH001703.1	GRF023	unpublished	Pdx_W1	Greece	Captive	Kalaentzis, Triantafyllidis, Gagavouzis
MH001704.1	GRF024	unpublished	Pdx_W1	Greece	Captive	Kalaentzis, Triantafyllidis, Gagavouzis
MH001705.1	GRF025	unpublished	Pdx_W1	Greece	Captive	Kalaentzis, Triantafyllidis, Gagavouzis
MH001706.1	GRF034	unpublished	Pdx_W1	Greece	Captive	Kalaentzis, Triantafyllidis, Gagavouzis
MH001707.1	GRF035	unpublished	Pdx_W1	Greece	Captive	Kalaentzis, Triantafyllidis, Gagavouzis
MH001708.1	GRF036	unpublished	Pdx_W1	Greece	Captive	Kalaentzis, Triantafyllidis, Gagavouzis
MH001709.1	GRF044	unpublished	Pdx_W1	Greece	Captive	Kalaentzis, Triantafyllidis, Gagavouzis
MH001710.1	GRF045	unpublished	Pdx_W1	Greece	Captive	Kalaentzis, Triantafyllidis, Gagavouzis
MH001711.1	GRF046	unpublished	Pdx_W1	Greece	Captive	Kalaentzis, Triantafyllidis, Gagavouzis
MH001712.1	GRF047	unpublished	Pdx_W1	Greece	Captive	Kalaentzis, Triantafyllidis, Gagavouzis
MH001713.1	GRF048	unpublished	Pdx_W1	Greece	Captive	Kalaentzis, Triantafyllidis, Gagavouzis

MH001714.1	GRF049	unpublished	Pdx_W1	Greece	Captive	Kalaentzis, Triantafyllidis, Gagavouzis
MH001715.1	GRF050	unpublished	Pdx_W1	Greece	Captive	Kalaentzis, Triantafyllidis, Gagavouzis
MH001716.1	GRF051	unpublished	Pdx_W1	Greece	Captive	Kalaentzis, Triantafyllidis, Gagavouzis
MH001717.1	GRF052	unpublished	Pdx_W1	Greece	Captive	Kalaentzis, Triantafyllidis, Gagavouzis
MH001718.1	GRU001	unpublished	Pdx_W1	Greece	Captive	Kalaentzis, Triantafyllidis, Gagavouzis
MH001719.1	GRU002	unpublished	Pdx_W1	Greece	Unknown origin	Kalaentzis, Triantafyllidis, Gagavouzis
MH001720.1	GRU003	W17	-	Greece	Unknown origin	Kalaentzis, Triantafyllidis, Gagavouzis
MH001721.1	GRU004	W17	-	Greece	Unknown origin	Kalaentzis, Triantafyllidis, Gagavouzis
MH001722.1	GRU005	unpublished	Pdx_W1	Greece	Unknown origin	Kalaentzis, Triantafyllidis, Gagavouzis
MH001723.1	GRU006	W17	-	Greece	Unknown origin	Kalaentzis, Triantafyllidis, Gagavouzis
MH001724.1	GRU007	unpublished	Pdx_W1	Greece	Wild	Kalaentzis, Triantafyllidis, Gagavouzis
MH001725.1	GRT001	unpublished	ME_E6_E14	Greece	Wild	Kalaentzis, Triantafyllidis, Gagavouzis
MH001726.1	GRT002	unpublished	ME_E6_E14	Greece	Wild	Kalaentzis, Triantafyllidis, Gagavouzis
MH001727.1	GRT003	unpublished	ME_E6_E14	Greece	Wild	Kalaentzis, Triantafyllidis, Gagavouzis
MH001728.1	GRT004	unpublished	ME_E6_E14	Greece	Wild	Kalaentzis, Triantafyllidis, Gagavouzis
MH001729.1	GRT005	unpublished	ME_E6_E14	Greece	Wild	Kalaentzis, Triantafyllidis, Gagavouzis
MH001730.1	GRG001	unpublished	ME_E6_E14	Greece	Wild	Kalaentzis, Triantafyllidis, Gagavouzis
MH001732.1	GRG005	unpublished	ME_E6_E14	Greece	Wild	Kalaentzis, Triantafyllidis, Gagavouzis
MH001734.1	GRG007	unpublished	ME_E6_E14	Greece	Wild	Kalaentzis, Triantafyllidis, Gagavouzis

MH001735.1	GRG008	unpublished	ME_E6_E14	Greece	Wild	Kalaentzis, Triantafyllidis, Gagavouzis
MH001743.1	GRK005	unpublished	ME_E6_E14	Greece	Wild	Kalaentzis, Triantafyllidis, Gagavouzis
MH001745.1	GRK007	unpublished	ME_E6_E14	Greece	Wild	Kalaentzis, Triantafyllidis, Gagavouzis
MH001746.1	GRK008	Pdx_GB_10	-	Greece	Wild	Kalaentzis, Triantafyllidis, Gagavouzis
MH001747.1	GRK009	Pdx_GB_10	-	Greece	Wild	Kalaentzis, Triantafyllidis, Gagavouzis
MH001748.1	GRK010	Pdx_GB_10	-	Greece	Wild	Kalaentzis, Triantafyllidis, Gagavouzis
MH001749.1	GRK011	Pdx_GB_10	-	Greece	Wild	Kalaentzis, Triantafyllidis, Gagavouzis
MH001750.1	GRK012	Pdx_GB_10	-	Greece	Wild	Kalaentzis, Triantafyllidis, Gagavouzis
MH001751.1	GRK013	Pdx_GB_10	-	Greece	Wild	Kalaentzis, Triantafyllidis, Gagavouzis
MH001752.1	GRD001	ME_E6_E14	-	Greece	Wild	Kalaentzis, Triantafyllidis, Gagavouzis
MN413491.1	B2	Pdx_GB_3	-	Italy	Captive	Pizzirani et al. 2020
MN413497.1	HET4	-	Pdx_W1	Italy	Captive	Pizzirani et al. 2020
MN413498.1	HET5	-	Pdx_W1	Italy	Captive	Pizzirani et al. 2020
MN413499.1	HET6	-	Pdx_W1	Italy	Captive	Pizzirani et al. 2020
MN413500.1	HET9	Pdx_GB_2	-	Italy	Captive	Pizzirani et al. 2020
MT410881.1	DM221	unpublished	Pdx_W1	Denmark	Unknown origin	DNAmark Project
MT649222.1	201720	W17	-	Italy	Captive	Pizzirani et al. 2020
MT649223.1	202420	-	Pdx_W1	Italy	Captive	Pizzirani et al. 2020
MT649224.1	20A320	-	Pdx_W2	Italy	Captive	Pizzirani et al. 2020
MT649225.1	20A1B20	-	Pdx_W1	Italy	Wild	Pizzirani et al. 2020
MT649226.1	20APP20	-	Pdx_W1	Italy	Captive	Pizzirani et al. 2020
OL513992.1	W31	Pdx_GB_2	-	Italy	Captive	Fontaneto et al. 2022
OL513993.1	W35	-	Pdx_W1	Italy	Captive	Fontaneto et al. 2022
OL513994.1	W34	-	Pdx_W1	Italy	Captive	Fontaneto et al. 2022
OL513995.1	W33	Pdx_GB_1U	-	Italy	Captive	Fontaneto et al. 2022

OL513996.1	W32		Pdx_W2	Italy	Captive	Fontaneto et al. 2022
-	-	ME_E6_E14	-	Greece	Wild	Liukkonen et al. 2002
-	-	E2	E2_E3_E4	Finland, Greece	Wild	Liukkonen et al. 2002
-	-	E3	E2_E3_E4	Finland	Wild	Liukkonen et al. 2002
-	-	E4	E2_E3_E4	Finland	Wild	Liukkonen et al. 2002
-	-	E5	-	Finland	Wild	Liukkonen et al. 2002
-	-	E7	-	Filande	Wild	Liukkonen et al. 2002
-	-	E8	-	Estonia	Wild	Liukkonen et al. 2002
-	-	E9	-	Sweden	Wild	Liukkonen et al. 2002
-	-	E10	-	Sweden	Wild	Liukkonen et al. 2002
-	-	E11	-	Russia	Wild	Liukkonen et al. 2002
-	-	E12	-	Kazakistan	Wild	Liukkonen et al. 2002
-	-	E13	-	Bulgaria	Wild	Liukkonen et al. 2002
-	-	E15	-	Sweden	Captive	Liukkonen et al. 2002
-	-	W3	Pdx_W2	Italy, France	Wild	Liukkonen et al. 2002
-	-	W2	W2_W28	Italy	Wild	Liukkonen et al. 2002
-	-	W28	E2_E3_E4	Finland	Captive	Liukkonen et al. 2002
-	-	W4	W4-W5-W6-W11-W16-W22-W29	France, Spain	Wild, Captive	Liukkonen et al. 2002
-	-	W5	W4-W5-W6-W11-W16-W22-W29	France	Wild	Liukkonen et al. 2002
-	-	W6	W4-W5-W6-W11-W16-W22-W29	France	Wild	Liukkonen et al. 2002
-	-	W11	W4-W5-W6-W11-W16-W22-W29	Bulgaria	Wild	Liukkonen et al. 2002
-	-	W16	W4-W5-W6-W11-W16-W22-W29	Sweden	Wild	Liukkonen et al. 2002
-	-	W22	W4-W5-W6-W11-W16-W22-W29	Italy, Poland	Tuscany Captive, Wild	Liukkonen et al. 2002
-	-	W29	W4-W5-W6-W11-W16-W22-W29	Sweden	Captive	Liukkonen et al. 2002
-	-	W7	-	France	Wild	Liukkonen et al. 2002
-	-	W8	-	France	Wild	Liukkonen et al. 2002
-	-	W9	W9_W10_W26	France	Wild	Liukkonen et al. 2002

-	-	W10	W9_W10_W26	France	Wild	Liukkonen et al. 2002
-	-	W26	W9_W10_W26	Finland	Captive	Liukkonen et al. 2002
-	-	W12	-	Poland	Wild	Liukkonen et al. 2002
-	-	W13	-	Poland	Wild	Liukkonen et al. 2002
-	W14	W14	-	Germany	Wild	Liukkonen et al. 2002
-	W15	W15	-	Germany	Wild	Liukkonen et al. 2002
-	W17	W17	-	Finland, Germany, Italy*, Latvia, Hungary*	Wild, Captive*	Liukkonen et al. 2002
-	W18	W18	-	England	Wild	Liukkonen et al. 2002
-	W19	W19	-	England	Wild	Liukkonen et al. 2002
-	W20	W20	-	Ireland	Wild	Liukkonen et al. 2002
-	W21	W21	-	Italy	Tuscany Captive	Liukkonen et al. 2002
-	W23	W23	-	Italy	Captive	Liukkonen et al. 2002
-	W24	W24	-	Italy	Captive	Liukkonen et al. 2002
-	W25	W25	-	Italy	Captive	Liukkonen et al. 2002
-	W27	W27	-	Finland	Captive	Liukkonen et al. 2002
-	W30	W30	-	Hungary	Captive	Liukkonen et al. 2002

Table S3

List of new haplotypes identified in this study, mutations and relative positions and GenBank Accession number . The position was determined in comparison with *Perdix perdix* mitochondrion complete genome, accession number NC_039843.1.

	175	176	184	188	189	196	211	217	219	220	236	240	242	243	244	245	259	294	302	308	309	310	GenBank Accession number
Pdx_W1	G	C	C	T	C	A	T	T	T	A	A	T	C	C	T	A	G	T	C	C	C	C	PQ299011
Pdx_W2	.	.	.	.	T	.	C	.	.	G	.	.	.	.	.	.	.	.	.	T	.	.	PQ299012
Pdx_W3	.	.	.	C	.	.	.	.	.	.	.	.	.	.	.	.	.	.	.	.	.	.	PQ299013
Pdx_W4	.	.	T	.	T	.	C	.	.	G	.	.	.	.	.	.	.	.	.	T	.	.	PQ299014
Pdx_W5	.	.	.	.	.	.	.	.	.	.	.	.	.	.	.	.	.	.	.	T	.	.	PQ299015
Pdx_W6	.	T	.	.	.	.	.	.	.	.	.	.	.	.	.	.	.	.	.	.	.	.	PQ299016
Pdx_W7	.	.	.	.	.	.	.	.	.	.	.	.	.	.	C	.	.	.	.	T	.	.	PQ299017
Pdx_W8	.	.	.	.	.	G	.	.	.	.	.	.	.	T	.	.	.	.	.	.	T	.	PQ299018
Pdx_W9	.	.	.	.	.	.	.	.	.	.	.	.	G	.	C	.	.	.	.	T	.	.	PQ299019
Pdx_W10	.	.	.	.	.	.	.	C	.	.	.	.	.	.	.	G	.	.	.	T	.	.	PQ299020
Pdx_W11	.	.	.	.	.	.	.	.	.	.	.	C	.	.	.	.	.	.	.	T	.	.	PQ299021
Pdx_W12	.	.	.	.	T	.	C	.	.	G	.	.	.	.	.	.	.	.	.	.	.	.	PQ299022
Pdx_W13	.	.	.	.	.	.	.	.	C	.	.	.	.	.	.	.	.	.	.	T	.	.	PQ299023
Pdx_W14	.	.	.	.	.	.	.	.	.	.	G	.	.	.	C	.	.	.	.	T	.	.	PQ299024
Pdx_E1	A	.	.	.	T	.	.	.	.	T	.	.	.	T	.	.	A	C	A	.	T	C	PQ299025

BOX S1

Insights into grey partridge subspecies

The different subspecies of grey partridge have been described, distinguished and classified on the sole basis of morphological traits without any genetic data in support; this is certainly indicative data but not exhaustive.

Presumably, this differentiation between local ecotypes, operated by natural selection, has an adaptive meaning linked to the different climatic conditions and to the vulnerability to predation. It evolved as a result of isolation and genetic local adaptations of populations to environmental conditions.

In any case, the differentiation between these subspecies appears decidedly limited, as far as the distinction between the various subspecies is concerned, they, in fact, have certain characteristic morphological traits with different frequencies, rather than exclusive characters. Western European varieties are characterized by a more rufous and brownish plumage, and Eastern ones by a greyer colouration.

Some authors recognize the existence of a further subspecies *P. p. Italica*, smaller than the Central European ones, presumably separated and differentiated from the others in the last glaciation. The acquisition of further knowledge is of particular importance, both to verify the possible survival of autochthonous populations in Italy to ensure their conservation, and to plan future reintroduction programs in the still suitable areas.

For the Italian peninsula, Hartert in 1917 described based on morphological and non-genetic traits, described a subspecies of grey partridge, the Italian grey partridge (*Pedix p. italica*) based on morphological traits, the subspecies was present above all in the Apennine hilly areas of central and southern Italy. The subspecies description was made considering specimens from the Chianti area of the early 1900s, currently kept in the American Museum of Natural History in New York. The author fixed an adult male captured at Badia Passignano, Chianti, on 20 January 1905 as the type specimen.

This study is based on the morphological analysis and the comparison between a sample of partridges of Italian origin and others of different origin: a large number of *Perdix p. perdix* and only nine *Perdix p. hispaniensis*.

If at first comparison the samples appeared indistinguishable, at a more careful analysis they showed marked differences in appearance. Concerning the nominal form, therefore, the italic one is described by Hartert as follows: The upper parts, in both sexes, are less dark and distinctly more brownish; throat and chest not so dark grey; horseshoe in the male and, if present in the female, not dark brown but chestnut as in *Perdix p. perdix*.

The male above all appears less rusty or reddish in the upper parts; the rump with predominantly dark brown strikethrough, instead of reddish it is overall appearance darker, with less presence of red-brown spots on the upper coverts of the wing. The female, with coarser colouring and marked by wide clear lines and specks, appears quite different from the female of *Perdix p. perdix*. Considering the scepticism of many authors and the doubts on the validity of the Italian subspecies, *P. p. italica* is of uncertain systematic value and too scarcely differentiated from the other subspecies, a study was carried out in Italy on museum samples.

In 1988 Violani et al. examined a consistent sample of grey partridges, 49 adult museum specimens, captured in Italy up to 1920 and marked as italic, additionally specimens belonging to other subspecies, especially the nominal form. Aiming to verify the recognizability of the *P. p. italica* as a distinguishable subspecies, they concluded that the original description made by Hartert was not based on objective and constant phenotypic differences, both as regards biometrics and colouration of plumage. The reasonable doubt on the existence of the italic subspecies is still unsolved. Genetics remains the only way to verify the validity at the subspecies level of the autochthonous Italian grey partridges (*Perdix perdix italica*) and, in association with the morphological and phenotypic description, distinguishing the subspecies. How far this population was genetically distant from Central European partridges has not yet been ascertained with certainty.

BOX S2

Letter of grey partridge samples request, sent to museum curators:

SUBJECT: Genetic identification of the Italian partridge *Perdix perdix italica*

To the head of the ornithological collection,

The National Institute for Wild Fauna "Alessandro Ghigi" has launched a genetic analysis program to identify the characteristics of the Italian partridge subspecies (*Perdix perdix italica*). To these purposes, we are collecting from museum specimens samples of partridges of Italian origin comparing subjects prior to 1920, a period in which genetic pollution due to reintroductions should have been minimal and subjects captured subsequently.

For each individual it is necessary to take 2 feathers complete with rachis and, if possible, a portion of 2 mm^3 of muscle tissue taken from the lower pad of a paw.

The sampling can be carried out by museum staff, who will be provided with the necessary equipment, or by one of our representatives. For further details, please refer to the attached sampling protocol.

We have identified in your museum some specimens of grey partridges suitable for inclusion in the project. We therefore require your authorization to proceed with the withdrawal.

The analyses will be carried out in 2004 and once completed a copy of the report will be sent to you.

We remain at your complete disposal for clarifications and further information.

Thanking you sincerely for your cooperation, we send you our best regards.

BOX S3

Sampling protocol provided to museums for samples collection of birds.

SAMPLING PROTOCOL MUSEUM BIRD SAMPLES

Biological samples taken from museum material are extremely delicate and susceptible to contamination. Therefore, the sampling must be carried out with utmost caution, wearing latex gloves, using disposable scalpels and working in conditions of maximum cleanliness to avoid contaminations.

The feathers must be taken from the undertail or from the lower remiges or primary or median coverts, preferably in two different positions and must be intact in the basal part, since it is the base of the rachis that will be used for DNA sampling. The epidermis and muscle tissue of the foot pad (callus) are an excellent source of DNA. In cases where it is possible, it will be sufficient to take a portion of 2 mm^3, incision of the epidermis with a disposable scalpel.

The material collected will be placed in special containers provided by INFS. Each sample will be labeled reporting the place and date of collection, as well as any data and notes reported. It would also be useful to have some photos of the specimens taken.

Samples have to be sent to:

Istituto Nazionale per la Fauna Selvatica, Via Cà Fornacetta, 9, cap 40064 Ozzano dell'Emilia (BO)
